# Identifying Chinese herbal medicine for premenstrual syndrome: implications from a nationwide database

**DOI:** 10.1186/1472-6882-14-206

**Published:** 2014-06-27

**Authors:** Hsing-Yu Chen, Ben-Shian Huang, Yi-Hsuan Lin, Irene H Su, Sien-Hung Yang, Jiun-Liang Chen, Jen-Wu Huang, Yu-Chun Chen

**Affiliations:** 1 Division of Chinese Internal Medicine, Center for Traditional Chinese Medicine, Chang Gung Memorial Hospital, Taoyuan, Taiwan; 2 Graduate Institute of Clinical Medical Sciences, College of Medicine, Chang Gung University, Taoyuan, Taiwan; 3 School of Traditional Chinese Medicine, College of Medicine, Chang Gung University, Taoyuan, Taiwan; 4 Department of Obstetrics and Gynecology, National Yang-Ming University Hospital, I-Lan, Taiwan; 5 School of Medicine, National Yang-Ming University, Taipei, Taiwan; 6 Department of Reproductive Medicine, University of California, La Jolla, San Diego, CA, USA; 7 Department of Surgery, National Yang-Ming University Hospital, I-Lan, Taiwan; 8 Department of Medical Research and Education, National Yang-Ming University Hospital, #152, Xin Min Rd, I-Lan 26042, Taiwan; 9 Faculty of Medicine, School of Medicine, National Yang-Ming University, Taipei, Taiwan; 10 Institute of Hospital and Health Care Administration, School of Medicine, National Yang-Ming University, Taipei, Taiwan

**Keywords:** Chinese herbal medicine, Premenstrual syndrome, Association rule mining, National health insurance research database, Social network analysis, Traditional Chinese medicine

## Abstract

**Background:**

Premenstrual syndrome (PMS) occurs in women during their reproductive age with a quite negative impact on their daily lives. Women with PMS experience a wide range of physical or psychological symptoms and seek treatment for them. Chinese herb medicine (CHM) is commonly used for PMS and the goal of this study is to investigate the prescription patterns of CHM for PMS by using a nationwide database.

**Methods:**

Prescriptions of CHM were obtained from two million beneficiaries randomly sampled from the National Health Insurance Research Database, a nationwide database in Taiwan. The ICD-9 code 625.4 was used to identify patients with PMS. Association rule mining and social network analysis were used to explore both the combinations and the core treatments for PMS.

**Results:**

During 1998-2011, a total of 14,312 CHM prescriptions for PMS were provided. Jia-Wei-Xiao-Yao-San (JWXYS) was the CHM which had the highest prevalence (37.5% of all prescriptions) and also the core of prescription network for PMS. For combination of two CHM, JWXYS with *Cyperus rotundus* L. was prescribed most frequently, 7.7% of all prescriptions, followed by JWXYS with *Leonurus heterophyllus* Sweet, 5.9%, and *Cyperus rotundus* L. with *Leonurus heterophyllus* Sweet, 5.6%.

**Conclusions:**

JWXYS-centered CHM combinations were most commonly prescribed for PMS. To the best of our knowledge, this is the first pharmaco-epidemiological study to review CHM treatments for PMS. However, the efficacy and safety of these commonly used CHM were still lacking. The results of this study provide valuable references for further clinical trials and bench studies.

## Background

Premenstrual syndrome (PMS) is a common gynecological disease among women in reproductive age. Most women experienced one or more physical, psychological or behavioral symptoms preceding or during menses; for some women, these symptoms severely disrupt their normal life [1]. The prevalence of PMS is high due to the various symptoms of PMS, such as food cravings, abdominal discomfort, headache, pain, depression, irritability and anxiety [2]. Around 75% of reproductive age women have mild symptoms and 20-30% of women have clinically significant PMS symptoms [3,4]. Additionally, 13-18% of women need treatment due to Premenstrual Dysphoric Disorder (PMDD), the severe form of PMS [5].

PMS symptoms have been attributed to fluctuations in levels of neurotransmitters in the central nervous system and/or cyclic hormonal changes. Central nervous system neurotransmitters such as serotonin (5-hydroxytryptamine) and γ-aminobutyric acid (GABA) appear to contribute to the pathogenesis of PMS [6-9]. Moreover, dynamic fluctuations in estradiol or progesterone in the late luteal phase may be another possible mechanism underlying PMS symptoms [10,11]. Accordingly, selective serotonin reuptake inhibitors (SSRIs) are the mainstay treatment of PMS, and hormone therapy to suppress ovulation is often used as well. In addition, other psychoactive drugs, such as alprazolam or buspirone, have been used for PMS with less effect [8,12].

These pharmacologic approaches are associated with side effects that obviously concern women with PMS. As a result, compliance in women with PMS treated with SSRIs is rather poor due to adverse drug effects, such as headache, weight gain, drowsiness, sexual dysfunction, and insomnia [13,14]. As well, prescriptions of alprazolam are limited due to drug dependence, intolerance or even abuse [8]. For these reasons, various types of traditional Chinese medicine (TCM) treatments, including acupuncture and Chinese herbal medicine (CHM), are used by women as an integrative approach to PMS [15-17]. Although TCM use is increasing yearly, information about CHM prescriptions used for women with PMS is still lacking [18].

CHM prescriptions are part of a rather complicated system and every prescription is made according to each patient’s TCM syndrome, or “Zheng” in Chinese, which is an overall summary of each patient’s physical signs or symptoms. Different diseases have their specific preponderance of TCM syndrome and the TCM syndrome varies from patient to patient [19]. Based on individuals’ diseases and TCM syndrome, different core treatments are prescribed and therefore it is feasible to discover the most important CHM and TCM understanding about a disease [20-22]. These findings are keys to further studies, especially new drug discovery, from the viewpoint of TCM system biology [23]. Application of association rule mining (ARM) and social network analysis (SNA) on a huge prescription database is a practical method for exploration of CHM core treatments and combinations, and several core treatments were successfully discovered in menopausal syndrome [24], primary dysmenorrhea [20], urticaria [21] and asthma [22].

The National Health Insurance Research Database (NHIRD), a nationwide health insurance claims database in Taiwan, is a comprehensive population-based tool that helpful to analyze the CHM prescription patterns [20-22,24]. The NHIRD is composed of all medical records, including ambulatory visits and hospitalization, reimbursed by the National Health Insurance (NHI) since 1995 [25]. Additionally, the NHI is unique in Taiwan, because both traditional Chinese medicine (CHM) and western medicine are equally reimbursed. Therefore, the option of using TCM or western medicine is completely unrestricted and largely dependent on the patient’s preference or habits. The quality in reimbursement is crucial to eliminate the bias on intervention selection. By using the NHIRD, the results of analysis on prescription patterns can be regarded as a consensus made by TCM doctors in Taiwan due to high coverage of the NHI, with over 99% of all Taiwanese population enrolled in the system [21,24].

We hypothesized that there should be core CHM treatment for PMS and this study aimed at discovering the core treatment patterns by applying ARM and SNA on the nationwide database. In addition to better understanding the viewpoint of TCM on PMS, further studies regarding efficacy, safety and mechanisms of action could be conducted based on the results of this work.

## Methods

### Data source

The NHIRD was used to obtain CHM prescriptions associated with the diagnosis of PMS. Two million individuals randomly sampled from the NHIRD and all medical claims of these individuals between 1998 and 2011 were extracted. This sample dataset is representative of the entire eligible Taiwanese population since no gender or age differences between sample and entire database were reported. The medical information contained in the NHIRD included patient’s birth date, gender, visit date, hospitalization date, medical services providers, treatments, managements, and the reasons for visits or hospitalization. The International Classification of Disease 9^th^ revision code (ICD-9) was used to identify the indications for medical services. Doctors may use up to three ICD-9 codes for every ambulatory visit and the first diagnosis was required to be the main reason for each visit. All TCM managements, including CHM, acupuncture and massage, were included.

### Study subjects

To recognize the PMS patients, ICD-9 code 625.4 (Premenstrual tension syndromes) was used and subject selection was performed by Chen YC and Chen HY, independently. PMS was characterized by periodical affective and somatic disorders, and it was associated with various symptoms [26,27]. Other mood disorders or menstrual diseases should be excluded, such as dysmenorrhea, endometriosis, or perimenopausal diseases, to make a definitive diagnosis; therefore, ambulatory visits with any diagnosis other than PMS were excluded. Additionally, only visits with a single diagnosis of PMS were included to reduce the influence of co-morbidities since TCM doctors usually treat patients by modifying a patients’ physical condition and by trying to treat all co-morbidities at once. Also, to eliminate the potential influence on choosing CHM, TCM visits with acupuncture and massage were excluded since these TCM modalities were thought to be adjuvant of CHM and may influence CHM prescription.

### Study variables

Frequencies, durations and dosage of CHM contained in prescriptions were the primary variables in this study to identify single agents and combinations of CHM commonly used for PMS. Two types of CHM are reimbursed by the NHI, single herb (SH) and herbal formula (HF), which follow fixed proportions of SH according to TCM classics. Quality control of CHM is assured as SH and HF are all produced as concentrated powders by Good Manufacturing Practice (GMP) pharmaceutical factories. In clinical practice, TCM doctor usually use several HF and/or SH in one prescription according to patient’s disease, TCM syndrome and associated symptoms. HF in prescriptions often serves as the core treatment for a disease and thus it is usually co-prescribed with other CHM according to TCM principles behind preparing prescriptions. The relationships between various CHM are typically quite complicated and a CHM network is commonly used to demonstrate the prescription patterns of CHM [20,28]. The frequency of connections among CHM is also assessed to verify the role of each CHM among network.

### Statistical analysis

Descriptive statistics were used to explore the prescription patterns of CHM for PMS. The prescription frequency, presented as prevalence, of the most commonly used CHM and their combinations, was analyzed. Moreover, the efficacy and potential underlying mechanisms of the commonly used CHM were thoroughly searched and reviewed. The English, Latin, Chinese names or corresponding Kampo medicine names were used in combination with “premenstrual syndrome” or “premenstrual dysphoric disorder” as the key words in searching references. Pubmed and Medline were both used as the target databases and the last assessed date was March 13, 2014. Chen HY, Lin YH, Yang SH and Chen JL were responsible for reviewing the searched references and determined the mechanisms of each listed CHM.

To explore the combinations of CHM, ARM and SNA were applied to illustrate the relationships between commonly used CHM. ARM is a statistical model widely used in medical research to examine associations between drugs or disease conditions, such as CHM prescription patterns for menopausal syndrome and analysis on co-morbidities of a disease [24,29]. Support and confidence factors are two key factors used in the ARM model to filter the significant associations among all CHM. Support factor was the prevalence of CHM combinations and confidence factor was similar to the conditional probability of a certain combination. For example, among two CHM, named CHM A and CHM B, support factor was presented as the probability (CHM A ∩ CHM B) and confidence factor was presented as the probability (CHM A ∩ CHM B)/probability (CHM A); only if both support and confidence factor were higher than the pre-set threshold was the association “CHM A with CHM B” considered significant. Significant associations were interpreted as “if CHM A was prescribed, CHM B was commonly used as well as an adjuvant”. Software R, version 2.15, and its statistical package “arule” was used to apply ARM model in this study. The threshold for support and confidence factors were set to 1% and 30%, and the threshold selected resulted from consensus among all co-authors of this work. The freeware NodeXL of SNA (http://nodexl.codeplex.com/) was used to illustrate the associations between commonly used CHM for PMS.

## Results

### Demographic characteristics of TCM users

Among two million representative dataset, a total of 5,668 patients were identified as having visited TCM doctors for PMS. During 1998-2011, a total of 14,312 visits were made by TCM visitors, and CHM was the most commonly used modality of TCM, accounting for 96.6% of all visits (13,820 visits). Additionally, TCM doctors commonly prescribed multiple CHM concomitantly; 5.4 CHM, including SH and/or HF, were used in on average in one prescription. Additionally, CHM was usually prescribed three times a day (89.0% of all prescriptions), followed by four times a day (8.4%).

### The most commonly used HF and SH

Among all prescriptions, Jia-Wei-Xiao-Yao-San (JWXYS) was the most commonly used CHM, accounting for 37.5% of all prescriptions, and it was also the most commonly used HF, followed by Dang-Gui-Shao-Yao-San (DGSYS) (2,064/14.9%) and Gui-Zhi-Fu-Ling-Wan (1,245/9.0%) (Table 1). Among single herbs, *Cyperus rotundus* L. was prescribed most frequently (2,485/18.0%), followed by *Leonurus heterophyllus* Sweet (2,097/15.2%) and *Corydalis yanhusuo* W. T. Wang (1,573/11.4%) (Table 2). Interestingly, although SH were usually used as an adjuvant to HF, *Cyperus rotundus* L. and *Leonurus heterophyllus* Sweet, both SH, were prescribed even more frequently than the HF DGSYS in this population. Moreover, the dose of commonly used HF was 2-3 times higher than SH, at approximately 4 gm/day versus less than 1.4 gm/day. (Tables 2 and 3).

**Table 1:** The top five most commonly used herbal formulas for premenstrual syndrome (N = 13,820)

**Herbal formula**	**Constituents**	**Indication of TCM syndrome**	**Instances**	**Dose (gm/day)**	**Duration (day)**	**Prevalence (%)**
Jia-Wei-Xiao-Yao-San (JWXYS)	*Paeonia lactiflora* Pall., *Bupleurum chinense* DC*.* DC*., Atractylodes macrocephala* Koidz.*, Poria cocos* (Schw.) Wolf*, Angelica sinensis* (Oliv.) Diels*., Mentha haplocalyx* Briq.*, Glycyrrhiza uralensis Fisch., Zingiber officinale* Rosc.*, Paeonia suffruticosa* Andr.*,* and *Gardenia jasminoides* Ellis.	Liver and spleen blood deficiency with heat transforming, liver qi stagnation	5,185	4.87	6.4	37.5
Dang-Gui-Shao-Yao-San	*Angelica sinensis (Oliv.) Diels., Ligusticum chuanxiong* Hort.*, Paeonia lactiflora* Pall.*, Atractylodes macrocephala* Koidz.*, Poria cocos* (Schw.) Wolf*,* and *Alisma plantago-aquatica* L. var. *Alisma orientale* Samuels	Liver blood deficiency and disharmony of Liver and Spleen	2,064	4.34	6.4	14.9
Gui-Zhi-Fu-Ling-Wan	*Cinnamomum cassia* Blume*, Poria cocos* (Schw.) Wolf, *Paeonia lactiflora* Pall.*, Paeonia suffruticosa* Andr.*, and Prunus persica* (L.) Batsch (or *Prunus davidiana* (Carr.) Franch.)	Blood stasis	1,245	3.87	6.3	9.0
Wen-Jing-Tang	*Evodia rutaecarpa* (Juss.) Benth.*,* (or *Evodia rutaecarpa* (Juss.) Benth. var*. officinalis* (Dode) Huang*, Evodia rutaecarpa* (Juss.) Benth. var. bodinieri (Dode) Huang)*, Cinnamomum cassia* Blume*, Angelica sinensis* (Oliv.) Diels.*, Paeonia lactiflora* Pall.*, Ligusticum chuanxiong* Hort*., Panax ginseng* C. A. Meyer*, Glycyrrhiza uralensis* Fisch.*, Equus asinus* L.*, Ophiopogon japonicus* Ker-Gawl.*, Pinellia ternata* (Thunb.) Breit*,* and *Zingiber officinale* Rosc.	Blood stasis and deficient cold	1,120	4.25	6.3	8.1
Shao-Fu-Zhu-Yu-Tang	*Foeniculum vulgare* Mill.*, Zingiber officinale* Rosc.*, Cinnamomum cassia* Blume*, Angelica sinensis* (Oliv.) Diels.*, Ligusticum chuanxiong* Hort.*, Paeonia lactiflora* Pall. *or Peaonia veitchii* Lynch*, Trogopterus xanthipes* Milne-Edwards *or Pteromys volans* L.	Qi stagnation and blood stasis	994	3.97	6.2	7.2

**Table 2:** The top ten most commonly used single herb for premenstrual syndrome (N = 13,820)

**Latin name**	**Indication of TCM syndrome**	**Instances**	**Dose (gm/day)**	**Duration (day)**	**Prevalence (%)**
*Cyperus rotundus* L.	Qi stagnation in liver	2,485	1.38	6.3	18.0
*Leonurus heterophyllus* Sweet	Blood stasis	2,097	1.33	6.3	15.2
*Corydalis yanhusuo* W. T. Wang	Qi stagnation and blood stasis	1,573	1.28	6.2	11.4
*Salvia miltiorrhiza* Bge.	Blood stasis	1,281	1.30	6.4	9.3
*Eucommia ulmoides* Oliv.	Kidney and liver deficiency	1,023	1.26	6.3	7.4
*Scutellaria baicalensis* Georgi	Dampness-heat	858	1.30	6.5	6.2
*Dipsacus asperoides* C. Y. Cheng at T. M. Ai	Blood stasis	644	1.17	6.4	4.7
*Cuscuta chinensis* Lam., or *Cuscuta japonica* Choisy	Kidney yang and yin deficiency	639	1.44	6.6	4.6
*Pueraria lobata* (Willd.) Ohwi *or Pueraria thomsonii* Benth.	External wind, fluid deficiency	616	1.33	6.4	4.5
*Paeonia suffruticosa* Andr.	Blood stasis and heat	605	1.43	6.6	4.4

**Table 3:** The top ten most commonly used two-herb combinations of Chinese herbal medicine (CHM) for premenstrual syndrome (N = 13,820)

**CHM A**		**CHM B**	**Instances**	**Prevalence (%)**
JWXYS	with	*Cyperus rotundus* L.	1,059	7.7
JWXYS	with	*Leonurus heterophyllus* Sweet	818	5.9
*Cyperus rotundus* L.	with	*Leonurus heterophyllus* Sweet	773	5.6
JWXYS	with	*Corydalis yanhusuo* W. T. Wang	564	4.1
*Cyperus rotundus* L.	with	*Corydalis yanhusuo* W. T. Wang	512	3.7
JWXYS	with	*Salvia miltiorrhiza* Bge.	467	3.4
*Cyperus rotundus* L.	with	*Salvia miltiorrhiza* Bge.	377	2.7
JWXYS	with	Gui-Zhi-Fu-Ling-Wan	375	2.7
JWXYS	with	*Scutellaria baicalensis* Georgi	262	1.9
JWXYS	with	*Curcuma longa* L. or *Curcuma aromatica* Salisb.	251	1.8

#### ***Two CHM in combinations for PMS***

JWXYS prescribed with *Cyperus rotundus* L. was the most frequently used two-CHM combination, accounting for 7.7% of all prescriptions (Table 3). JWXYS with *Leonurus heterophyllus* Sweet (818/5.9%) and *Cyperus rotundus* L. with *Leonurus heterophyllus* Sweet (773/5.6%) were the second and third the most frequently prescribed combinations. “HF with SH” were the most often used combinations (80%), rather than “SH with SH” (20%) concept in TCM.

### Core treatment for PMS

**Figure 1:**
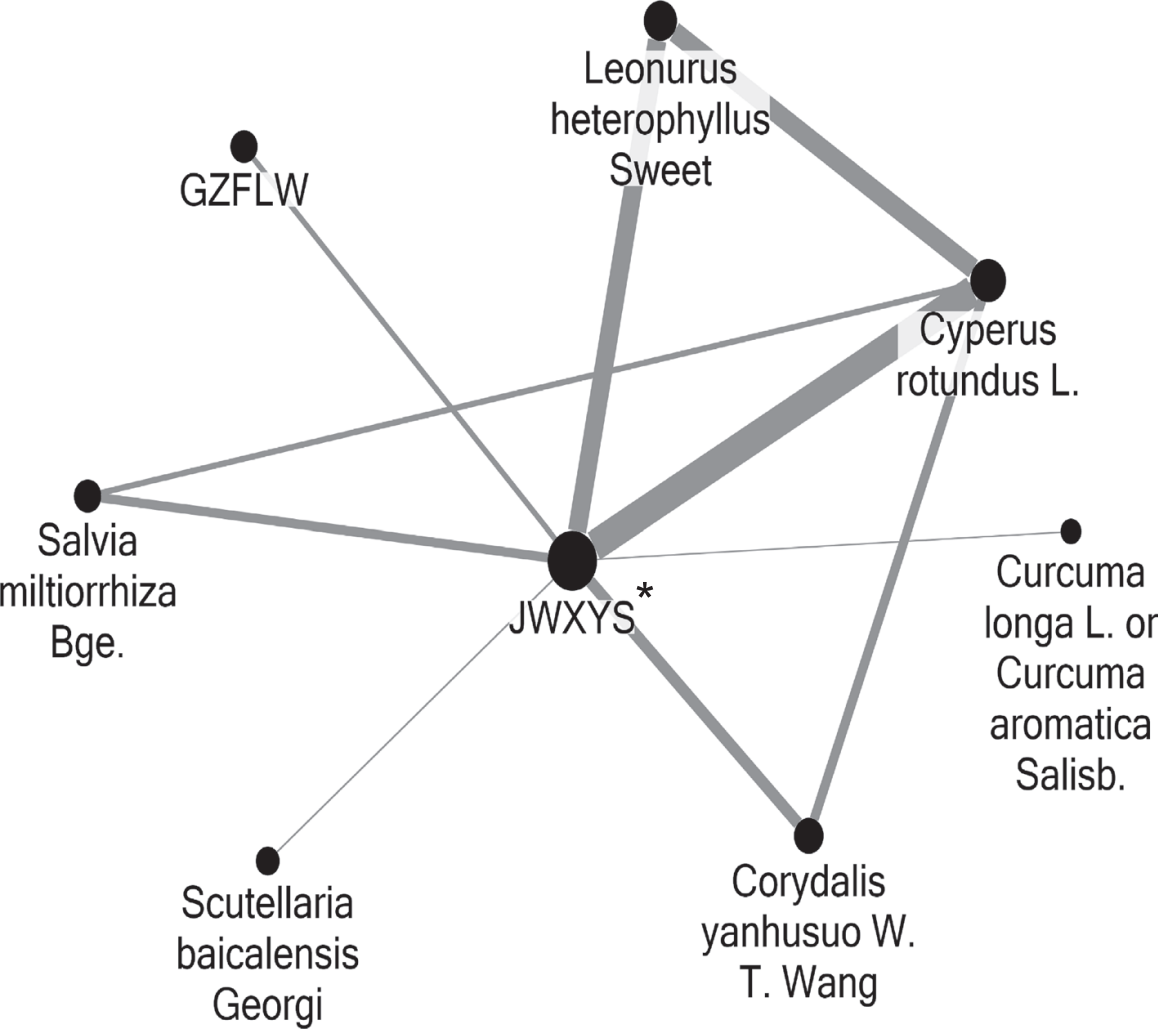
**Social network analysis on associations between commonly used CHM (top 10).** Abbreviations: JWXYS: Jia-Wei-Xiao-Yao-San; GZFLW: Gui-Zhi-Fu-Ling-Wan. *JWXYS was the center of the CHM network prescribed for PMS and became the core treatment for PMS. **Larger circle represents higher prevalence, and thicker connection line represents more common combination.

JWXYS was the core treatment for PMS because it accounted for the highest prevalence among all CHM and 80% of the most commonly used two-CHM combinations. Additionally, the central role of JWXYS can be found in the network of commonly used CHM (Figure 1). Other CHM scattered peripherally represented their adjuvant action of PMS.

## Discussion

The key finding of this study is that JWXYS was the core treatment for PMS and certain CHM, such as *Cyperus rotundus* L. and *Leonurus heterophyllus* Sweet, were frequently prescribed with JWXYS. This is the first pharmaco-epidemiological study about CHM for PMS. Among all CHM used for PMS, only JWXYS was examined for its efficacy and included in systematic review [15,17]. The effectiveness and safety of other commonly used CHM, especially when they are combined with JWXYS, have not been studied yet, and CHM included in latest systematic review are not exactly commonly used in daily practice [17]. The results of this study provide ideal candidate for further studies as we strive to achieve the goal of establishing evidence-based medicine viewpoints on CHM.

JWXYS has been reported to have anxiolytic and anti-depression effects in animal models although its effectiveness on PMS remains unclear (Table 4). From TCM’s viewpoint, JWXYS, which was developed about a thousand years ago in the TCM classic of prescriptions, is used to treat liver and spleen blood deficiency with heat transforming, which fairly represents mood disorder, gastrointestinal dysfunction and menstrual problems due to endocrine disorder. Therefore, it is frequently used to treat primary dysmenorrhea [20], menopausal syndrome [24], depression [30] and functional dyspepsia [31]. The high prevalence of psychological discomfort and gastrointestinal problems among PMS women in Taiwan may be the reason for the leading role of JWXYS in all CHM [32].

**Table 4:** Possible pharmacological mechanisms of Chinese herbal medicine (CHM) used for premenstrual syndrome

**Name**	**Ingredients**	**Possible mechanisms of extract of SH**
**Single herb (SH)**		
*Cyperus rotundus* L.	Hydroalcoholic extract	Anti-oxidative effect [33]
	Essential oil	Anti-oxidative effect [34]
*Leonurus heterophyllus* Sweet	Methanol extract	Anti-oxidative effect [35]
*Corydalis yanhusuo* W. T. Wang	Tetrahydropalmatine	Anxiolytic effect [36]
		Analgesic effect [37]
*Salvia miltiorrhiza* Bge.	Miltirone	Anxiolytic effect [38]
	Salvianolic acid B	Anti-depressant effect [39]
		Analgesic effect [40]
		Anti-oxidative effect [41]
	Tanshinone IIA	Analgesic effect [42]
*Eucommia ulmoides* Oliv.	Water extract	Anti-oxidative effect [43,44]
**Herbal formula (HF)**		
Jia-Wei-Xiao-Yao-San (JWSYS)		Anti-depressant effect on animal model [45,46]
		IL-6 related anti-depressant effect on menopausal women with mood disorder [47]
		Anxiolytic effect on animal model [48]
		Reduced proinflammatory cytokine [49]
Dang-Gui-Shao-Yao-San		Anti-depressant effect on animal model [50]
		Anti-oxidative effect in vitro [51]
Wen-Jing-Tang		Anti-depressant effect for menopausal women [52]

In addition to this core treatment, several CHM were found to be significantly associated with core treatments, and thus a network of CHM prescribed for PMS can be established (Figure 1). These combinations of CHM were valuable guides for clinical practice since TCM doctors usually use several CHM in one prescription (5.4 CHM on average). This finding was expected as it has its foundation in TCM teachings and with potential pharmacological effects of CHM, CHM prescriptions are made according to an individual’s constitution, namely bian-zheng-lun-zhi, which means the identification of TCM syndrome and treatment. It is reasonable since human physiology is complex and multiple strategies to treat PMS may be needed. Interestingly, anti-oxidative effects are reported extensively among the CHM surrounding JWXYS [33,35,41,43], which may complement JWXYS since JWXYS lacks anti-oxidative effect. The anti-oxidative effects exclusively found in SH strongly imply the importance of oxidative stress on PMS, such as *Cyperus rotundus* L. and *Leonurus heterophyllus* Sweet (Table 4). To date, only one study based on 41 patients discussed the potential association between PMS and this imbalance of oxidants/anti-oxidants [53]. Thus, the essential role of anti-oxidation therapy for PMS will require further studies in order to be defined properly. This finding represents the merit of using ARM and social network analysis to analyze the relations between CHM, and not just focusing on the use of a single CHM.

Furthermore, same treatments for different diseases, another feature of CHM prescription, could be found when comparing the prescription patterns among PMS, primary dysmenorrhea and menopausal syndrome [20]. For example, JWXYS was also commonly used for mood disorder in menopausal women [24] and DGSYS was frequently used for primary dysmenorrhea and PMS [20]. This concept is quite different from western medicine which often treats PMS after excluding other diagnoses such as dysmenorrhea. Therefore, the treatment principle considerably differs: analgesia for primary dysmenorrhea and mood modifying for PMS. On TCM’s viewpoint, prescriptions are made not only according to “disease” but also TCM syndrome, “zheng”, and therefore it is not uncommon to prescribe same formula to various diseases if the same TCM syndrome is diagnosed [54].

“Qi stagnation and blood stasis” seemed the most prevalent TCM syndrome for PMS. Although it was not feasible to perform detailed analysis on TCM syndrome of PMS since TCM syndrome was not recorded in the NHIRD, the distribution of TCM syndrome could be shown when summarizing the indications of commonly used CHM [21,55]. Ten out of top 15 commonly used CHM are indicated to manage “qi stagnation” or “blood stasis” (Tables 1 and 2) and JWXYS is effective in affective disorder caused by liver qi stagnation syndrome [56]. Qi stagnation usually represented lesion distension or pain due to impeded qi circulation and mood disorders, such as depression or anxiety and depression was highly associated with liver qi stagnation [57]. Further, severe qi stagnation may obstruct blood flow to cause blood stasis, but blood stasis may aggravate qi stagnation conversely. This TCM syndrome and pathogenesis reflected the concept about PMS: mood disorders (qi stagnation) and menstrual discomfort (blood stasis) are highly related. For this reason, regulation on menstrual cycle is needed to treat PMS for TCM doctors and that is why many CHM other than JWXYS are added into one prescription for PMS.

This study explored the core formula, JWXYS, and important combinations of CHM for PMS. This result is especially crucial in searching for candidates that are suitable for clinical or bench studies for PMS since CHM prescriptions are usually different from textbooks themselves [58]. The merit of this study is that the subject selection is done from a nationwide database and thus possible selection and referral bias can be eliminated. Nonetheless, there are a number of limitations to consider. First, the counts of prescriptions for PMS might be underestimated since only visits with a single diagnosis of PMS were used to identify the study population in this study. Nevertheless, it is believed that the use of a single diagnosis is helpful in eliminating unnecessary bias, especially when PMS is a syndrome with various presentations and the impact from co-morbidities may be considerable. Second, the validity of ICD-9 code for the diagnosis of PMS should be considered. In Taiwan, the first diagnosis code of each ambulatory visit represents the main reason for the visit. As physicians and institutions are penalized for fraudulent coding, monthly internal monitoring are usually performed to validate diagnoses. This internal monitoring theoretically improves the accuracy of the database. Therefore, we believe the diagnostic codes are specific and the registration bias can be minimized. Third, the efficacy of therapies was not determined in this study. Despite the fact that the nationwide database contains large amounts of prescription data, there are no data on PMS severities, laboratory or image examinations since these data are not required in the electronic medical records. Consequently, the effectiveness of CHM for PMS is unable to be assessed by using this database.

## Conclusions

The high prevalence and role of the core treatment JWXYS for PMS is shown in this study. Also, the combinations of CHM with JWXYS, such as *Cyperus rotundus* L. and *Leonurus heterophyllus* Sweet, demonstrate the potential effectiveness of anti-oxidant agents on PMS. Based on these results, further studies about CHM for PMS are warranted both in bench research and clinical trials to examine the effectiveness of these therapies.

### Ethical approval

This study was approved by the Institutional Review Board (IRB) of Chang Gung Memorial Foundation, Taipei, Taiwan (IRB: 101-3604B).

## Abbreviations

PMS: Premenstrual syndrome; CHM: Chinese herbal medicine, (JWXYS) Jia-Wei-Xiao-Yao-San; ICD-9: The International classification of disease, 9th revision; PMDD: Premenstrual dysphoric disorder; GABA: γ-aminobutyric acid; ARM: Association rule mining; NHIRD: The National Health Insurance Research Database; NHI: The National Health Insurance; SNA: Social network analysis; TCM: Traditional Chinese medicine.

## Competing interests

The authors report that they have no conflicts of interest to declare.

## Authors’ contributions

HC and BH were responsible for study design, result interpretation and drafted the manuscript. HC also participated in data management. IS revised the manuscript and provided expert opinions as a professional gynecologist. YL, SY, JC, and JH participated in study design and result interpretation. In addition, HC, YL, SY and JC were the investigators to review and summarize the pharmacologic mechanisms of CHM. YC participated in data management, statistics analysis and helped to manuscript writing. All authors read and approved the final version of this manuscript.

## Authors’ information

HC, YL, SY and JC are TCM doctors of Division of Chinese Internal Medicine, Center for Traditional Chinese Medicine, Chang Gung Memorial Hospital. In Taiwan, it has one of the largest TCM departments of all medical centers. HC is responsible for teaching evidence-based medicine of TCM in this department. By collaborating with YC, who is the head of Department of Medical Research and Education, National Yang-Ming University Hospital, several epidemiology studies about TCM are conducted. We are interested in how and why CHM are prescribed for patients for individual diseases and we believe this information is critical for TCM on the viewpoints of evidence-based medicine. These results are beneficial to study candidate selection and results of further clinical trials or bench studies will be much more adherent to clinical practice.

## Pre-publication history

The pre-publication history for this paper can be accessed here:

http://www.biomedcentral.com/1472-6882/14/206/prepub
